# The basal to total insulin ratio in outpatients with diabetes on basal-bolus regimen

**DOI:** 10.1007/s40200-018-0358-2

**Published:** 2018-10-01

**Authors:** Elena Castellano, R. Attanasio, V. A. Giagulli, A. Boriano, M. Terzolo, E. Papini, E. Guastamacchia, S. Monti, A. Aglialoro, D. Agrimi, E. Ansaldi, A. C. Babini, A. Blatto, D. Brancato, C. Casile, S. Cassibba, C. Crescenti, M. L. De Feo, A. Del Prete, O. Disoteo, F. Ermetici, V. Fiore, A. Fusco, D. Gioia, A. Grassi, D. Gullo, F. Lo Pomo, A. Miceli, M. Nizzoli, M. Pellegrino, B. Pirali, C. Santini, S. Settembrini, E. Tortato, V. Triggiani, A. Vacirca, G. Borretta, all on behalf of Associazione Medici Endocrinologi (AME)

**Affiliations:** 1 Department of Endocrinology, Diabetes and Metabolism, Santa Croce and Carle Hospital, Via Michele Coppino 26, 12100 Cuneo, Italy; 2 Endocrinology Service, Galeazzi Institute, IRCCS, Milan, Italy; 3 Outpatient Clinic for Endocrinology and Metabolic Diseases, Conversano Hospital, Conversano, Italy; 4 Medical Physics Department, Santa Croce and Carle Hospital, Cuneo, Italy; 5 Internal Medicine, Department of Clinical and Biological Sciences, San Luigi Hospital, Orbassano, Italy; 6 Department of Endocrinology and Metabolism, Regina Apostolorum Hospital, Albano Laziale, Italy; 7 Department of Medicine-Section of Internal Medicine, Geriatrics, Endocrinology and Rare Diseases, Bari, Italy; 8 Endocrinology, Department of Clinical and Molecular Medicine, Sant’Andrea Hospital, Rome, Italy; 9 Metabolism and Diabetes Unit, San Martino Hospital, Genoa, Italy; 10 District Hospital, Azienda Sanitaria Locale, Brindisi, Italy; 11 Department of Endocrinology and Diabetes, Santissimi Antonio e Biagio Hospital, Alessandria, Italy; 12 Medical Division, Rimini Hospital, Rimini, Italy; 13 Department of Endocrinology, Maria Vittoria Hospital, Torino, Italy; 14 Department of Internal Medicine and Diabetology, Hospital of Partinico, Partinico, Italy; 15 Internal Medicine Department, Papardo Hospital, Messina, Italy; 16 Endocrinology and Diabetes, Papa Giovanni XXIII Hospital, Bergamo, Italy; 17 Department of Endocrinology and Diabetes, San Giovanni di Dio Hospital, Florence, Italy; 18 Endocrinology Unit, Careggi Hospital, Florence, Italy; 19 Outpatient Clinic for Diabetes, Azienda Sanitaria Locale, Civita Castellana, Italy; 20 Diabetology Department, Niguarda Hospital, Milan, Italy; 21 Endocrinology and Metabolism, IRCCS Policlinico San Donato, San Donato Milanese, Italy; 22 Angelucci Hospital, Subiaco, Italy; 23 Antidiabetic Center AID, Garibaldi Hospital, Naples, Italy; 24 Department of Endocrinology, Villa Sofia Hospital, Palermo, Italy; 25 Division of Endocrinology, Mauriziano Umberto I Hospital, Torino, Italy; 26 Endocrinology, Department of Clinical and Experimental Medicine, Garibaldi-Nesima Medical Center, Catania, Italy; 27 Division of Endocrinology, San Carlo Hospital, Potenza, Italy; 28 Department of Endocrinology, Morgagni Hospital, Forlì, Italy; 29 Unit of Internal Medicine, Humanitas Mater Domini, Castellanza, Italy; 30 Department of Endocrinology and Diabetology, Bufalini Hospital, Cesena, Italy; 31 Diabetology Service, Azienda Sanitaria Locale Na 1, Naples, Italy; 32 Diabetology Service, Augusto Murri Hospital, Fermo, Italy; 33 Department of Internal Medicine, Imola Hospital, Imola, Italy

**Keywords:** Insulin therapy, Hypoglycemia, Basal bolus, Type 1 diabetes, Type 2 diabetes

## Abstract

**Objective:**

To evaluate the basal/total ratio of daily insulin dose (b/T) in outpatients with diabetes type 1 (DM1) and type 2 (DM2) on basal-bolus regimen, by investigating whether there is a relationship with HbA1c and episodes of hypoglycemia.

**Methods:**

Multicentric, observational, cross-sectional study in Italy. Adult DM1 (*n* = 476) and DM2 (*n* = 541) outpatients, with eGFR >30 mL/min/1.73 m^2^, on a basal-bolus regimen for at least six months, were recruited from 31 Italian Diabetes services between March and September 2016. Clinicaltrials.govID: NCT03489031.

**Results:**

Total daily insulin dose was significantly higher in DM2 patients (52.3 ± 22.5 vs. 46 ± 20.9 U/day), but this difference disappeared when insulin doses were normalized for body weight. The b/T ratio was lower than 0.50 in both groups: 0.46 ± 0.14 in DM1 and 0.43 ± 0.15 in DM2 patients (*p* = 0.0011). The b/T was significantly higher in the patients taking metformin in both groups, and significantly different according to the type of basal insulin (Degludec, 0.48 in DM1 and 0.44 in DM2; Glargine, 0.44 in DM1 and 0.43 in DM2; Detemir, 0.45 in DM1 and 0.39 in DM2). The b/T ratio was not correlated in either group to HbA1c or incidence of hypoglycemia (<40 mg/dL, or requiring caregiver intervention, in the last three months). In the multivariate analysis, metformin use and age were independent predictors of the b/T ratio in both DM1 and DM2 patients, while the type of basal insulin was an independent predictor only in DM1.

**Conclusion:**

The b/T ratio was independent of glycemic control and incidence of hypoglycemia.

**Electronic supplementary material:**

The online version of this article (10.1007/s40200-018-0358-2) contains supplementary material, which is available to authorized users.

## Introduction

Insulin is the most effective drug for the treatment of diabetes [1]. Basal-bolus regimen is the most recommended approach for insulin-treated patients with diabetes, as it reflects the physiological insulin secretion and improves glycemic control more safely than other insulin regimens [2, 3]. The basal-bolus insulin regimen is the starting treatment for individuals with type 1 (DM1) diabetes, whereas in patients with diabetes type 2 (DM2), it is the final step when an optimal glycemic control is not obtained with other antidiabetic drugs (OADs), or these are contraindicated.

Current guidelines [1, 3] provide few recommendations regarding the distribution of insulin doses for the basal-bolus regimen. In a hospital setting, 60–80% of the total (i.e. basal plus bolus units) daily insulin dose is recommended in DM1 and DM2 patients transitioning to outpatient subcutaneous insulin [1]. The American Association of Clinical Endocrinologists (AACE) guidelines also recommend starting subcutaneous insulin treatment in DM2 patients by halving the total daily dose between basal and prandial fractions, the latter divided into three doses before meals [3]. In fact, the subdivision of total daily insulin dose in real life is not evidence based and its association with glycemic control is still unresolved.

Our multicentric, observational, cross-sectional study aimed to evaluate the basal/total ratio of daily insulin dose (b/T) in a large series of DM1 and DM2 patients by looking for a possible relationship with the glycemic control (as evaluated by HbA1c) and the occurrence of hypoglycemia.

## Patients and methods

### Design and protocol

From March 2016 to September 2016, we performed an observational, cross-sectional, multicenter study in outpatients with DM1 and DM2 diabetes. The study was commissioned by the Italian Association of Clinical Endocrinologists (Associazione Medici Endocrinologi - AME) and approved by the Ethical Committee of Cuneo Hospital (BASAL Study— OSS 001/2016 – rif ENDO 30). The Clinicaltrials.gov ID is NCT03489031. The research was open to all specialists taking care of patients with diabetes in Italy.

The primary endpoint was glycemic control, evaluated by HbA1c levels, according to b/T.

Secondary endpoints were occurrence of major hypoglycemic episodes according to b/T; differences between patients with DM1 and DM2.

An ad hoc form was developed and used to record all medical findings. The form was emailed to all participating centers that then emailed or faxed it back to our data manager. Data were checked for accuracy.

The following data were required: age, gender, body weight and height, country of origin, type of diabetes and its duration, units of basal and total (basal plus prandial) daily insulin, type of basal insulin (Glargine, Detemir or Degludec), use and dosage of metformin, and number of major hypoglycemic episodes (<40 mg/dL or requiring caregivers intervention) in the last three months. Serum creatinine and HbA1c levels obtained within the previous two months were also required.

### Patients

Each participating center recruited between 20 and 40 outpatients with diabetes. Inclusion criteria were as follows: adult outpatients (≥20 and ≤ 80 years) with DM1 or DM2, on basal-bolus insulin regimen (basal bedtime insulin, i.e. Glargine, Detemir or Degludec, plus at least two prandial insulin shots with insulin short acting analogs) for at least six months, usually assuming three daily meals, and capable of informed consent.

Exclusion criteria: pregnancy or breast-feeding, severe liver or renal failure (eGFR <30 mL/min/1.73 m^2^), use of OADs (only metformin was allowed); hospitalization for any cause, glucocorticoid or oncologic treatment, or patients who had performed Ramadan in the last six months.

Patients gave their informed consent, and the Institutional Review Board of each participating center approved the study. All procedures followed were in accordance with the ethical standards of the Helsinki Declaration of 1964, as revised in 2013.

### Methods

All biochemical parameters were analyzed in the laboratories of each participating center. HbA1c was assayed by the ion-exchange HPLC method. Serum creatinine levels were assayed by automated analysis using a colorimetric method. eGFR was calculated using the CKD-EPI formula [4], namely:

$$ \mathrm{eGFR}={141}^{\ast }\ \min\ \left(\mathrm{SCr}/\mathrm{k},1\right)\ {\upalpha}^{\ast }\ \max\ \left(\mathrm{SCr}/\mathrm{k},1\right)-{1.209}^{\ast }\ 0.99{3}^{\ast}\mathrm{Age}\ \left[\ast 1.018\ \mathrm{if}\ \mathrm{women}\ \right]\ \left[\ast 1.159\ \mathrm{if}\ \mathrm{black}\right], $$

where SCr represents serum creatinine (in mg/dL), k represents 0.7 for women and 0.9 for men, α represents −0.329 for women and − 0.411 for men, min represents the minimum of SCr/k or 1, and max represents the maximum of SCr/k or 1.

Patient body mass index (BMI) was calculated as the body weight in kilograms divided by the square of the body height in meters.

### Statistical analysis

Variables were preliminarily tested for normal distribution with the Shapiro-Wilks W test. Data were expressed as mean ± SD, or median and interquartile range (IQR) as appropriate. Continuous variables with non-normal and normal distribution were analyzed by ANOVA for repeated measures followed by the Tukey test, completely randomized ANOVA followed by the Games-Howell test, Mann-Whitney U test and t test for unpaired samples, respectively, as appropriate. Differences in categorical variables were analyzed by χ^2^ or Fisher’s test, as appropriate.

Setting b/T as the dependent variable, two separate sets of linear regressions were performed. The first set was univariate; the second included all the variables whose β-coefficient was significant in the univariate analysis. In the case of multiple independent variables, a multicollinearity test was performed. Variables were rejected from the analysis if there was a variance inflation factor greater than 2.

The level of statistical significance was set at *p* ≤ 0.05 with the Bonferroni correction.

Calculations were performed using SPSS (IBM SPSS Statistics – version 21).

## Results

The study was performed in 31 Italian centers, equally distributed among northern, central and southern Italy. We enrolled 1017 patients (aged 56.9 ± 17.7 years), male 57% and female 43%, nearly evenly subdivided between DM1 and DM2, namely 47 and 53%, respectively. Table 1 summarizes the demographic, clinical and biochemical data of the whole study group. DM1 patients were significantly younger, slimmer and with a longer disease duration than DM2 patients. In addition, DM1 patients had a significantly higher eGFR and better glycemic control, but a higher incidence of hypoglycemia.

**Table 1:** Demographic and clinical data

	All (*n* = 1017)	DM1 (*n* = 476)	DM2 (*n* = 541)	*p*
Males/Females (%)	57/43	58.4/41.6	55.9/44.1	0.52
Age (years)	56.9 ± 17.7	43.8 ± 15.2	67.9 ± 9.8	**0.00001**
Country of origin (Italy/other) (%)	95.2/4.8	94.9/5.1	95.4/4.6	0.1
BMI (kg/m^2^)	28.2 ± 6.7	25.3 ± 4.3	30.8 ± 7.4	**0.00001**
Disease duration (years)	18.2 ± 11	19.2 ± 12.5	17.3 ± 9.4	**0.0059**
Basal insulin (U/day)	21.5 ± 10.4	20.9 ± 10.2	22.1 ± 10.6	0.067
Total insulin (U/day)	49.3 ± 22.0	46 ± 20.9	52.3 ± 22.5	**0.00001**
Total insulin daily dose (U/kg)	0.63 ± 0.26	0.63 ± 0.26	0.63 ± 0.26	1
b/T ratio	0.45 ± 0.15	0.46 ± 0.14	0.43 ± 0.15	**0.0011**
HbA1c (%)	7.96 ± 1.4	7.78 ± 1.2	8.1 ± 1.4	**0.0001**
HbA1c (mmol/mol)	63.6 ± 14.9	61.5 ± 13.6	65.4 ± 15.6	**0.0001**
Metformin users (%)	28.1	13.9	40.0	**0.0001**
Mean metformin daily dose (mg)	1723 ± 695	1411 ± 653	1823 ± 671	**0.0001**
Creatinine (mg/dL)	0.93 ± 0.3	0.96 ± 0.31	0.90 ± 0.25	**0.0007**
eGFR (mL/min/1.73 m^2^)	89 ± 35	94 ± 36	85 ± 34	**0.00001**
Patients with major hypoglycemic episodes (%)	15.1	19.2	11.5	**0.0015**
Patients with > 3 hypoglycemic episodes (%)	12.3	20.7	0	**0.0005**
Type of basal insulin (%)				
• Glargine	59.8	49.5	68.8	**0.00001**
• Detemir	7.9	4.5	10.9	
•Degludec	32.3	46	20.3	

Demographic and clinical data of the whole series. *P* value is comparing DM1 and DM2 patients

Abbreviations: *BMI* body mass index; *b/t*, basal to total insulin ratio; *eGFR* estimated glomerular filtration rate

Bold points to statistically significant difference

Metformin was used in a greater proportion of DM2 patients than in DM1 patients (40% vs. 13.9%), at higher dosages (Table 1). The relative proportion of patients using the different types of basal insulin, namely Degludec, Detemir and Glargine, was significantly different between DM1 and DM2 patients (*p* = 0.00001). Total daily insulin dose was significantly higher in DM2 patients, but this difference disappeared if insulin doses were normalized for body weight.

The total insulin daily dose was significantly higher in patients with eGFR >60 mL/min/1.73 m^2^ than in those with impaired renal function, both in DM1 (54.63 ± 21.61 U vs. 44.33 ± 21 U; *p* = 0.0001) and in DM2 patients (47.05 ± 20.34 U vs. 34.86 ± 13.27 U; *p* = 0.00001).

The b/T ratio was lower than 0.50 in both groups, and significantly different between DM1 and DM2 patients (0.46 ± 0.14 vs. 0.43 ± 0.15, respectively, *p* = 0.011). The b/T was significantly higher in patients taking metformin, in both groups: DM1, 0.52 ± 0.11 vs. 0.45 ± 0.11 (p = 0.00001) and DM2, 0.45 ± 0.11 vs. 0.41 ± 0.11 (p = 0.00001).

In the univariate analysis, the b/T ratio was not related to HbA1c or to the incidence of hypoglycemia, in both DM1 and DM2 patients. In both groups, the b/T ratio was significantly directly correlated to metformin use and inversely correlated to age (Tables 2 and 3). The b/T ratio was significantly influenced by the type of basal insulin, both in DM1 (*p* = 0.003) and in DM2 (*p* = 0.021) patients. It was higher with Degludec (0.48 in DM1 and 0.44 in DM2), intermediate with Glargine (0.44 in DM1 and 0.43 in DM2), and lower with Detemir (0.45 in DM1 and 0.39 in DM2).

**Table 2:** Correlations of b/T in DM1 patients (n = 476) at univariate analysis

	r	P
Age	**−0.136**	**0.003**
Sex		0.665
BMI		0.488
Country of origin		0.806
Diabetes duration		0.357
Type of basal insulin		**0.003**
HbA1c		0.743
eGFR		0.06
Hypoglycemia occurrence		0.808
Metformin use		**<0.0001**

Correlations of b/T with demographical and clinical parameters in T1 patients at univariate analysis. R coefficient value is provided for non-categorical variables with *p* < 0.05

Abbreviations: *BMI* body mass index; *eGFR* estimated glomerular filtration rate

Bold points to statistically significant difference

**Table 3:** Correlations of b/T in DM2 patients (n = 541) at univariate analysis

	r	P
Age	**−0.114**	**0.003**
Sex		0.178
BMI		0.963
Country of origin		0.825
Diabetes duration		0.053
Type of basal insulin		**0.021**
HbA1c		0.255
eGFR		0.145
Hypoglycemia occurrence		0.875
Metformin use		**0.0004**

Correlations of b/T with demographical and clinical parameters in T2 patients at univariate analysis. R coefficient value is provided for non-categorical variables with p < 0.05

Abbreviations: *BMI* body mass index; *b/T* basal to total insulin ratio; *eGFR* estimated glomerular filtration rate

Bold points to statistically significant difference

In the multivariate regression, age, type of insulin and use of metformin were significantly associated with b/T in DM1 (ß = −0.122, *p* = 0.007; ß = 0.135, p = 0.003; ß = 0.232, *p* < 0.0001; respectively); only age and use of metformin maintained a statistical relationship with b/T in DM2 patients (ß = −0.099, *p* = 0.026; ß = 0.134, p = 0.003; respectively).

## Discussion

This large multicentric observational study highlights that in outpatients with diabetes on basal-bolus insulin regimen, the amount of basal insulin is usually less than 50% of the total daily dose. The b/T ratio was significantly different between DM1 and DM2 patients, i.e. 46 and 43%, respectively. Moreover, neither the glycemic control nor the occurrence of hypoglycemia seemed to be linked to the b/T ratio. Conversely, the b/T ratio appeared to be significantly correlated to age and metformin co-administration.

The basal-bolus regimen is widely recommended by the current guidelines for the treatment of patients with DM1 and DM2. In fact this regimen improves glycemic control and reduces hospital complications compared to other conventional therapeutic approaches [2]. Basal-bolus insulin therapy provides a physiological approach to the treatment of patients with diabetes inasmuch as it covers both basal (i.e., overnight fasting and between-meals) and prandial (i.e., glucose excursions above basal levels at mealtimes) insulin needs [5].

Despite the biologically sound foundations of the basal-bolus schedule, the literature data are scant regarding its practical application in real life. For the transition from IV to SC insulin administration in inpatients with diabetes, current guidelines suggest administering 60–80% of the total daily insulin infused dose as basal insulin [1]. The AACE guidelines also recommend that SC insulin treatment in DM2 patients be started by halving the total daily dose between basal and prandial fractions, the latter divided into three doses before meals. The subsequent titration is based on mealtime and fasting glycemic targets [3]. However, evidence is still lacking regarding the practical implementation of basal-bolus insulin regimen in long-term diabetes.

To the best of our knowledge, this is the first study evaluating the b/T ratio in a large series of patients with DM1 and DM2 referred to departments specialized in the care of patients with diabetes. Our findings show that, in real life in Italy, basal insulin is usually less than 50% of the total daily dose. It is unknown whether in other populations the starting insulin dose suggested by guidelines is applied and maintained in the long-term. However, it is likely that the characteristics of the Mediterranean diet [6], usually followed by the Italian population, play a major role in our findings.

The b/T ratio was significantly higher in DM1 than DM2 patients. A variety of factors could account for this finding. Firstly, DM1 patients produce no insulin [7], and higher basal insulin doses are thus necessary in order to cover the insulin needs of overnight fasting. In addition, DM2 patients are on average fatter and with worse glycemic control than DM1 patients. This is thus likely to lead to a higher requirement of prandial insulin in DM2 patients. It is well established that obesity and a sedentary lifestyle, typical of DM2 patients, result in a loss of insulin sensitivity [8], with greater insulin needs at mealtimes. In addition, physical activity has been shown to improve insulin sensitivity and is associated with moderately lower postprandial glucose levels [9, 10].

Finally, the role played by the type of long-acting insulin used cannot be excluded. The b/T ratio was significantly related to the type of basal insulin used. In fact, basal insulin analogs have different pharmacodynamic and pharmacokinetic characteristics, with a clinical impact on timing, flexibility and dose [11]. Analogs with longer duration of action have a prolonged and more consistent glucose-lowering effect [11], thus implying the reduced need for prandial insulin. This mechanism might explain the higher b/T ratio found in patients treated with Degludec.

The b/T ratio was unrelated to the incidence of hypoglycemia in both diabetic groups. Note that only patients treated with long-acting basal analogs were included in our study. International diabetes guidelines [1, 3] all recommend using long-acting basal analogs instead of NPH, to reduce the risk of symptomatic and nocturnal hypoglycemia [12–14], in terms of a comparable glycemic control [15, 16]. The most recently marketed Degludec, which is a long-acting insulin analog with a prolonged half-life and ultra-flat kinetics [11], is the best choice in DM1 patients requiring lower HbA1c targets with the consequent potential higher risk of hypoglycemia [11, 17].

Hypoglycemia was significantly more frequent in DM1 than in DM2 patients. This could be due to the stricter control of the glycemic target achieved by DM1 patients. In fact, diabetic microvascular complications are reduced in patients achieving a target of HbA1c <7% (53 mmol/mol) [18]. However, further lowering of the HbA1c goal is associated with a substantially increased risk of hypoglycemia, thus outweighing the potential benefits in terms of microvascular complications [19].

We found a direct correlation between the b/T ratio and the use of metformin both in DM1 and DM2 patients. Metformin acts primarily by decreasing the endogenous glucose production, in particular the hepatic gluconeogenesis after a meal [20, 21]. It is conceivable that its use would enable the prandial insulin dose to be reduced.

The daily percentage of basal insulin decreases in patients suffering from renal impairment. It is well known that the kidney plays a pivotal role in insulin metabolism and excretion. Insulin is filtered at the glomerulus and almost completely reabsorbed and degraded by cells lining the proximal convoluted tubules. This mechanism accounts for 50–60% of the renal uptake of insulin, while the remaining 40–50% is removed from the post-glomerular peritubular capillaries [22]. In patients with renal impairment, the insulin half-life is markedly prolonged, particularly for basal insulin [23]. These observations account for the drop in insulin requirements of diabetic patients with reduced eGFR, as well as the significantly lower daily insulin doses in the patients of this series with renal impairment.

We found an inverse correlation between age and the b/T ratio. It is known that ageing promotes insulin resistance in DM1 [24] and in DM2 patients [25], due to the overexpression of fat mass. The fat mass, above all its visceral component, co-regulates fasting plasma glucose [26] and insulin sensitivity [27] in DM1 and DM2 patients of both sexes [28, 29]. All these factors could account for the higher need for prandial insulin in the elderly.

The strengths of this study are inherent in its design, reflecting the current therapeutic approach to diabetes mellitus in real life practice.

As for the limitations, this study focused on a homogeneous population, in terms of ethnicity and lifestyle, from all regions in Italy. These findings cannot therefore be generalized to patients with diabetes from other countries and ethnic groups. We acknowledge that information on carbohydrate consumption, carbohydrate counting education and physical activity are lacking. Moreover, the residual β cell function was not evaluated. This could play a role, in particular in DM2 patients, in influencing insulin requirements and the distribution between prandial and basal insulin. Moreover, it is to take into account that in real world, DM2 obese patients are rarely managed with basal bolus insulin regimen and metformin alone.

Finally, the putative relationship between the b/T ratio and the analyzed outcomes must be interpreted with caution, because the design of the study was cross-sectional and not longitudinal.

In conclusion, in a large series of Italian outpatients with diabetes treated with an insulin basal-bolus regimen, the b/T ratio was lower than 50% and significantly higher in DM1 than in DM2 patients. The b/T ratio was independent of glycemic control and the incidence of hypoglycemia, while it was correlated to metformin use and age in both DM1 and DM2 patients. Further longitudinal studies are necessary in order to define a causal association between the b/T ratio and the efficacy and safety of insulin treatment.

## Electronic supplementary material

ESM 1 (DOC 26 kb)

## Abbreviations

b/T: basal to total daily insulin dose ratio
CI: confidence intervals
HbA1c: glycated hemoglobin
eGFR: estimated glomerular filtration rate
HPLC: high pressure liquid chromatography
OADs: other antidiabetic drugs
SD: standard deviation
DM1: type 1 diabetes
DM2: type 2 diabetes
