# Correction to: The basal to total insulin ratio in outpatients with diabetes on basal-bolus regimen

**DOI:** 10.1007/s40200-018-0370-6

**Published:** 2018-12-17

**Authors:** Elena Castellano, R. Attanasio, V. A. Giagulli, A. Boriano, M. Terzolo, E. Papini, E. Guastamacchia, S. Monti, A. Aglialoro, D. Agrimi, E. Ansaldi, A. C. Babini, A. Blatto, D. Brancato, C. Casile, S. Cassibba, C. Crescenti, M. L. De Feo, A. Del Prete, O. Disoteo, F. Ermetici, V. Fiore, A. Fusco, D. Gioia, A. Grassi, D. Gullo, F. Lo Pomo, A. Miceli, M. Nizzoli, M. Pellegrino, B. Pirali, C. Santini, S. Settembrini, E. Tortato, V. Triggiani, A. Vacirca, G. Borretta, all on behalf of Associazione Medici Endocrinologi (AME)

**Affiliations:** 1 Department of Endocrinology, Diabetes and Metabolism, Santa Croce and Carle Hospital, Via Michele Coppino 26, 12100 Cuneo, Italy; 2 Endocrinology Service, Galeazzi Institute, IRCCS, Milan, Italy; 3 Outpatient Clinic for Endocrinology and Metabolic Diseases, Conversano Hospital, Conversano, Italy; 4 Medical Physics Department, Santa Croce and Carle Hospital, Cuneo, Italy; 5 Internal Medicine, Department of Clinical and Biological Sciences, San Luigi Hospital, Orbassano, Italy; 6 Department of Endocrinology and Metabolism, Regina Apostolorum Hospital, Albano Laziale, Italy; 7 Department of Medicine-Section of Internal Medicine, Geriatrics, Endocrinology and Rare Diseases, Bari, Italy; 8 Endocrinology, Department of Clinical and Molecular Medicine, Sant’Andrea Hospital, Rome, Italy; 9 Metabolism and Diabetes Unit, San Martino Hospital, Genoa, Italy; 10 District Hospital, Azienda Sanitaria Locale, Brindisi, Italy; 11 Department of Endocrinology and Diabetes, Santissimi Antonio e Biagio Hospital, Alessandria, Italy; 12 Medical Division, Rimini Hospital, Rimini, Italy; 13 Department of Endocrinology, Maria Vittoria Hospital, Torino, Italy; 14 Department of Internal Medicine and Diabetology, Hospital of Partinico, Partinico, Italy; 15 Internal Medicine Department, Papardo Hospital, Messina, Italy; 16 Endocrinology and Diabetes, Papa Giovanni XXIII Hospital, Bergamo, Italy; 17 Department of Endocrinology and Diabetes, San Giovanni di Dio Hospital, Florence, Italy; 18 Endocrinology Unit, Careggi Hospital, Florence, Italy; 19 Outpatient Clinic for Diabetes, Azienda Sanitaria Locale, Civita Castellana, Italy; 20 Diabetology Department, Niguarda Hospital, Milan, Italy; 21 Endocrinology and Metabolism, IRCCS Policlinico San Donato, San Donato Milanese, Italy; 22 Angelucci Hospital, Subiaco, Italy; 23 Antidiabetic Center AID, Garibaldi Hospital, Naples, Italy; 24 Department of Endocrinology, Villa Sofia Hospital, Palermo, Italy; 25 Division of Endocrinology, Mauriziano Umberto I Hospital, Torino, Italy; 26 Endocrinology, Department of Clinical and Experimental Medicine, Garibaldi-Nesima Medical Center, Catania, Italy; 27 Division of Endocrinology, San Carlo Hospital, Potenza, Italy; 28 Department of Endocrinology, Morgagni Hospital, Forlì, Italy; 29 Unit of Internal Medicine, Humanitas Mater Domini, Castellanza, Italy; 30 Department of Endocrinology and Diabetology, Bufalini Hospital, Cesena, Italy; 31 Diabetology Service, Azienda Sanitaria Locale Na 1, Naples, Italy; 32 Diabetology Service, Augusto Murri Hospital, Fermo, Italy; 33 Department of Internal Medicine, Imola Hospital, Imola, Italy

**Correction to: J Diabetes Metab Disord**

10.1007/s40200-018-0358-2

The article *The basal to total insulin ratio in outpatients with diabetes on basal-bolus regimen*, was originally published electronically on the publisher’s internet portal (currently SpringerLink) on [1st October 2018] without open access. With the author(s)’ decision to opt for Open Choice the copyright of the article changed on [17th November 2018] to © The Author(s) 2018 and the article is forthwith distributed under the terms of the Creative Commons Attribution 4.0 International License (http://creativecommons.org/licenses/by/4.0/), which permits use, duplication, adaptation, distribution and reproduction in any medium or format, as long as you give appropriate credit to the original author(s) and the source, provide a link to the Creative Commons license and indicate if changes were made.

The original article was corrected.

**Publisher’s Note** Springer Nature remains neutral with regard to jurisdictional claims in published maps and institutional affiliations.

