# Supplementary material for: The basal to total insulin ratio in outpatients with diabetes on basal-bolus regimen
Source: J Diabetes Metab Disord. 2018 Oct 1;17(2):393–9. doi: 10.1007/s40200-018-0358-2 (PMC6405380; doi:10.1007/s40200-018-0358-2)
Supplement: Supplementary file 1 — (DOC 26 kb) [file 40200_2018_358_MOESM1_ESM.doc]

Why carry out this study?

- Current guidelines provide few indications regarding the distribution of insulin doses for the basal-bolus regimen.
- The subdivision of total daily insulin dose in real life is not evidence based and its association with glycemic control is still unresolved
- Our study aimed to evaluate the basal/total ratio of daily insulin dose in a large series of T1 and T2 patients by looking for a possible relationship with the glycemic control and the occurrence of hypoglycemia.

What was learned from the study? What were the study outcomes/conclusions?

- Our study highlights that in diabetic outpatients on basal-bolus insulin regimen, the amount of basal insulin is usually less than 50% of the total daily dose.
- Neither the glycemic control nor the occurrence of hypoglycemia seemed to be linked to the b/T ratio.
- Conversely, the b/T ratio appeared to be significantly correlated to age and metformin co-administration.
